# Supplementary material for: Seminoma and Embryonal Carcinoma Footprints Identified by Analysis of Integrated Genome-Wide Epigenetic and Expression Profiles of Germ Cell Cancer Cell Lines
Source: PLoS One. 2014 Jun 2;9(6):e98330. doi: 10.1371/journal.pone.0098330 (PMC4041891; doi:10.1371/journal.pone.0098330)
Supplement: File S1 — ZIP file containing DMRforPairs output for significant regions. Please start from the html files. (ZIP) [file pone.0098330.s008.zip › figures/37215.pdf]

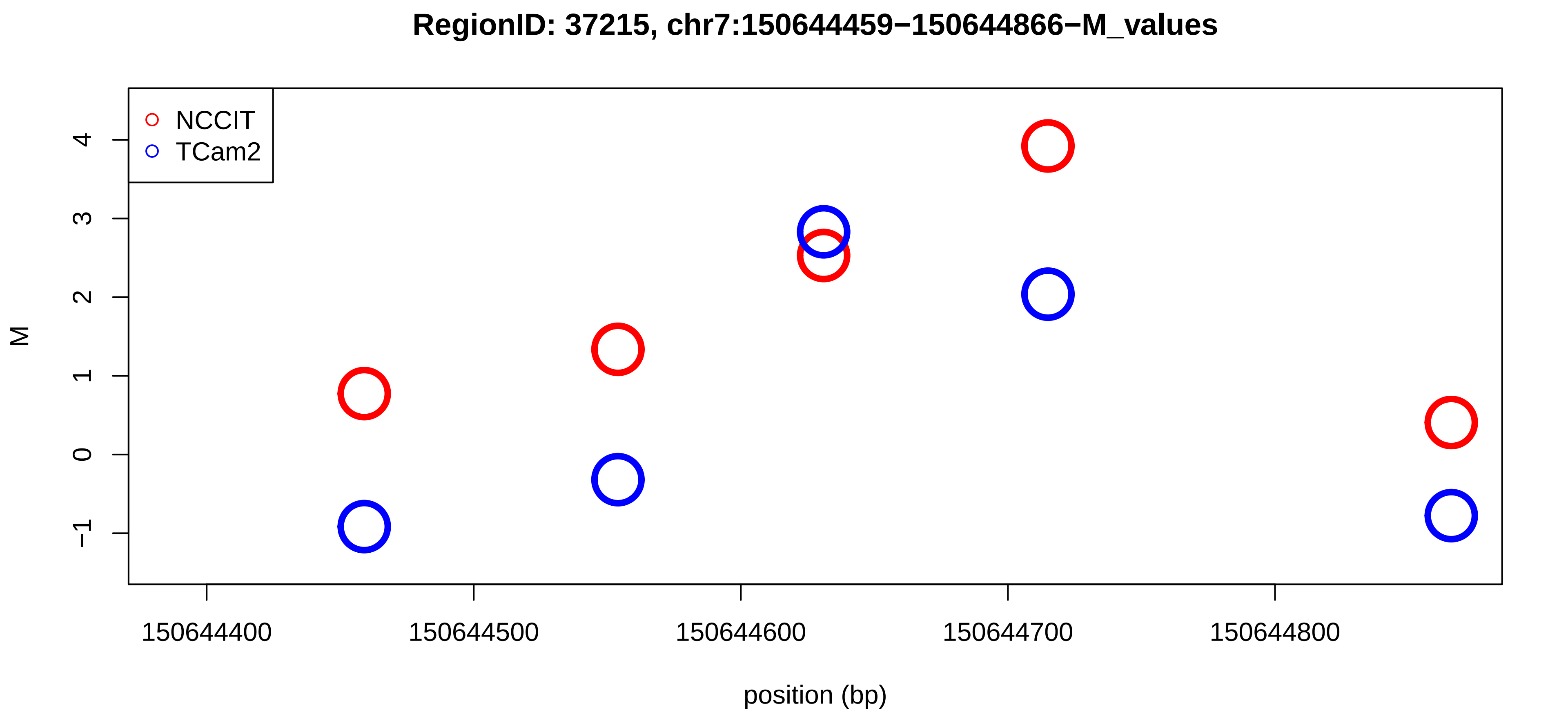
RegionID: 37215, chr7:150644459−150644866−M_values
NCCIT
TCam2
M
position (bp)
150644400
150644500
150644600
150644700
150644800
−1
0
1
2
3
4

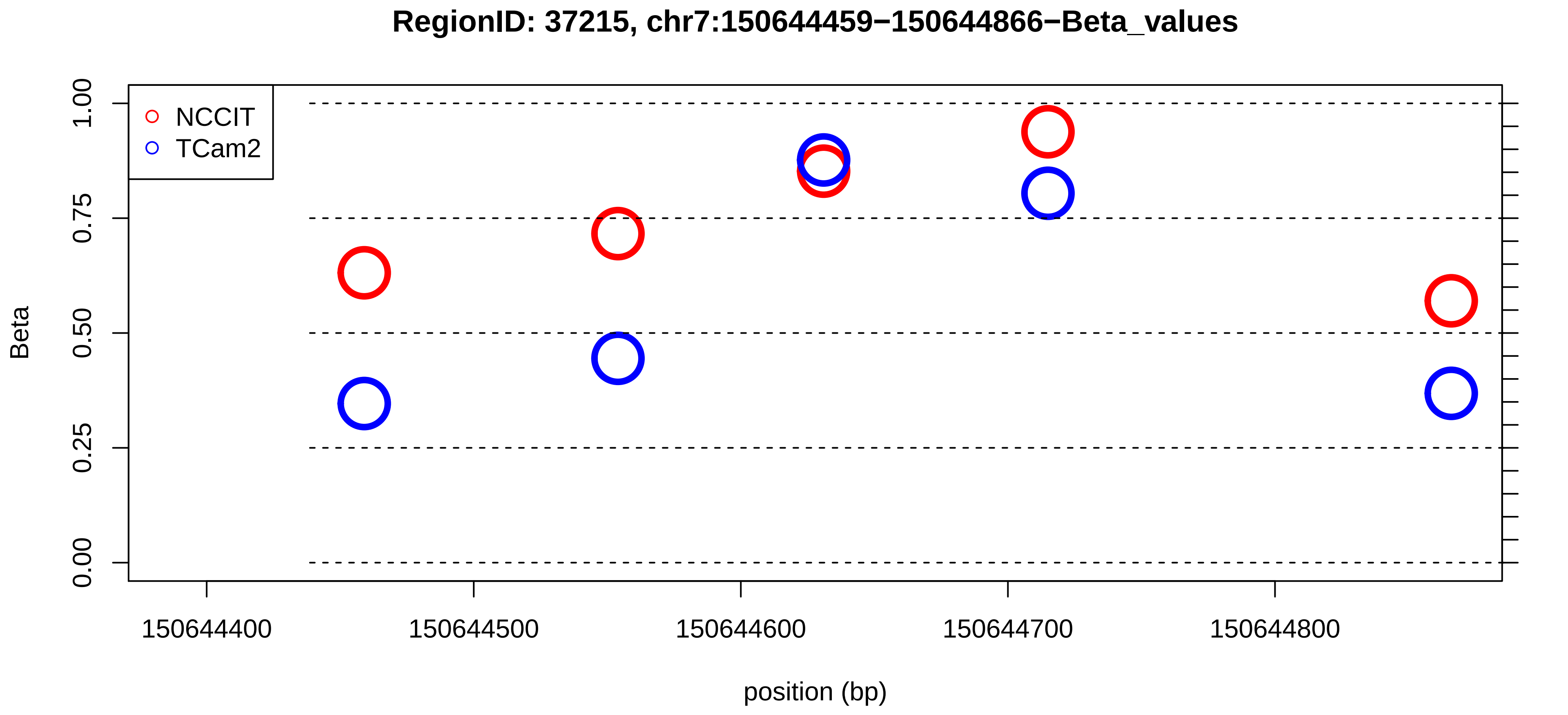
RegionID: 37215, chr7:150644459−150644866−Beta_values
NCCIT
TCam2
Beta
position (bp)
150644400
150644500
150644600
150644700
150644800
0.00
0.25
0.50
0.75
1.00
